# Venesection and resolution of erythrocytosis are not associated with reduced thrombotic risk in secondary and idiopathic polycythaemia: Results from a dual centre, 5‐year retrospective study

**DOI:** 10.1111/bjh.20235

**Published:** 2025-06-30

**Authors:** B. D. Maybury, E. Jongman, C. Morgan, K. Sheikh, J. Prasangika, M. Asif, M. Bisp, C. Lam, F. Noor, S. A. Y. Abbas, H. Dhunya, R. Lloyd, M. H. A. Chowdhury, P. Rathnayake, M. Aquil, W. Butterfield, T. Jha, P. Dyer, R. J. Buka, P. L. R. Nicolson, HaemSTAR Collaborators, Zoha Mansoor, Farhan Patel, Sanober Sanober, Rahul Ghandi, Eman Hassan

**Affiliations:** ^1^ Department of Haematology University Hospitals Birmingham, NHS Foundation Trust, Queen Elizabeth Hospital Birmingham UK; ^2^ HaemSTAR Birmingham UK; ^3^ Department of Haematology University Hospital of North Midlands NHS Trust, Royal Stoke University Hospital Stoke‐on‐Trent UK; ^4^ Department of Cardiovascular Sciences, College of Medicine and Health University of Birmingham Birmingham UK; ^5^ School of Medicine, Keele University Newcastle‐under‐Lyme UK

**Keywords:** diagnostic haematology, erythrocytosis, polycythaemia, thrombosis, venesection

To the Editor,

Secondary polycythaemia is 10 times more common than polycythaemia vera (PV),^1^ but evidence to guide its management is very sparse. We report the results of a retrospective audit of diagnosis and management, including 5‐year outcome data, of 206 patients with secondary polycythaemia from two large hospital trusts. Investigation and management varied widely, but therapeutic venesection was not associated with a faster or greater reduction in haematocrit (Hct) after diagnosis, nor was it associated with a lower risk of thrombosis.

Polycythaemia is defined by the British Society for Haematology (BSH) as a persistent haemoglobin (Hb) >165 g/L or Hct >0.48 in females and Hb >185 g/L or Hct >0.52 in males.^2^ The World Health Organization (WHO) defines it less stringently as Hb >160 g/L or Hct >48% in females and Hb >165 g/L or Hct >49% in males.^3^ Secondary causes of polycythaemia include high erythropoietin (EPO) states, such as hypoxic pulmonary disease (HPD), cyanotic congenital heart disease (CHD), EPO‐secreting tumours and the use of anabolic steroids. An apparent polycythaemia can result from reduction in plasma volume due to diuretic use, alcohol excess and possibly sodium/glucose cotransporter 2 (SGLT2) inhibitors.^4^, ^5^ Congenital polycythaemia has a variety of well‐characterised genetic causes relating to erythropoietin regulation or haemoglobinopathy.^6^ Idiopathic polycythaemia was historically a diagnosis of exclusion; however, recent studies have identified germline and somatic genetic causes in some of these patients.^7^

In PV, there is clear evidence for both increased thrombotic risk and a reduction in this risk by aspirin, pharmacological cytoreduction and/or venesection to keep Hct <0.45.^8^, ^9^ In idiopathic polycythaemia, there is weak evidence of increased thrombotic risk,^10^ and for secondary polycythaemia, specifically, there is an absence of evidence informing thrombotic risk, although at a population level, higher Hct is associated with thrombosis.^11^ There is no good quality evidence to guide the management of idiopathic or secondary polycythaemia. BSH recommendations are to consider venesection in some circumstances.^12^

However, venesection itself is not without risk: It can induce iron deficiency, syncope and nerve injury.^13^ Indeed, in congenital heart disease, venesection is harmful and increases stroke risk, and so is only recommended in those with severe hyperviscosity symptoms and Hct 0.65–0.70.^14^ In HPD, evidence for venesection is limited to small studies of transient physiological outcomes, and observational studies show that patients with polycythaemia survive longer than those with anaemia. In HPD, polycythaemia is a compensatory response to hypoxia and correcting it may be harmful.

In the absence of good quality evidence, there is significant variation in the management of secondary polycythaemia, whether or not to venesect, and threshold/target Hct.^15^ A randomised trial could define the effects of venesection in secondary polycythaemia, but to design a study with known statistical power, systematic data on patient outcomes are needed.

Using the HaemSTAR network, we performed a retrospective audit of compliance with BSH polycythaemia guidelines^12^ across two large academic hospital trusts in the United Kingdom. The audit was approved by both trusts. Key inclusion criteria were a negative test for *JAK2* V617F between 1 January 2014 and 31 December 2018; no diagnosis of myeloproliferative neoplasm; no prior investigation for myeloproliferative neoplasm; Hb or Hct above the WHO thresholds for polycythaemia. We collected baseline, treatment and outcome data over at least 5 years of follow‐up. The primary outcomes were compliance with BSH guidelines on the investigation and management of secondary/idiopathic polycythaemia. Exploratory outcomes included rates of thrombosis and overall survival. Paired *t*‐tests were used to compare Hct over time, and log‐rank and Cox proportional hazards were used for time‐to‐event analyses. Landmark analyses and modelling venesection exposure as a time‐dependent variable were used to reduce the risk of immortal time bias. Full details on data collection and statistical analyses performed are in the Supporting Information.

Of 2360 *JAK2* V617F‐negative patients screened, we collected data on 206 patients with BSH‐defined secondary polycythaemia who had 5 years of follow‐up data (Figure 1A). The median age at diagnosis was 54 years, 70% of patients were male and 90% were white Caucasian. The median Hct at diagnosis was 0.51 (female) and 0.54 (male). 26% and 49% of patients had definite and probable secondary polycythaemia respectively. 12% had idiopathic polycythaemia and 10% had apparent polycythaemia. Contributory factors included smoking (56% of cases), hazardous alcohol consumption (23%), HPD (19%), sleep apnoea (11%), androgen use (5%) and CHD (1.5%), with a median of 1 (interquartile range: 1–2) contributory factors per patient. 18% of patients were tested for mutations in exon 12 of *JAK2* and were negative. Baseline variables and outcome data are summarised in Table 1.

**FIGURE 1:**
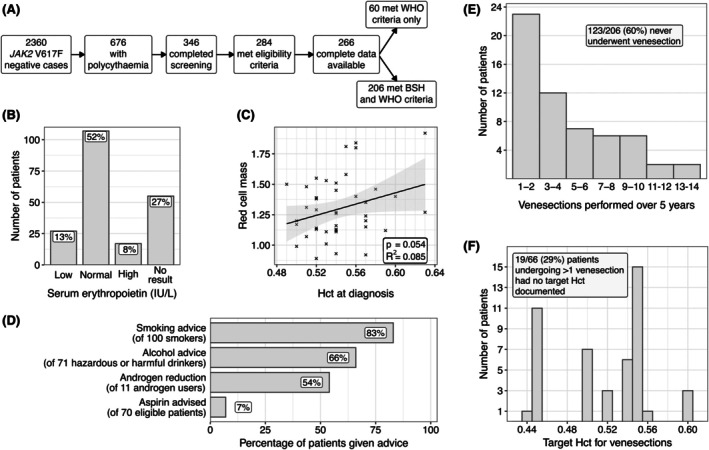
Investigation and management of secondary polycythaemia. (A) Disposition of cases screened. (B) Serum erythropoietin results, relative to the local laboratory's reference range. (C) Haematocrit (Hct) at diagnosis plotted against red cell mass measured by nuclear red cell dilution assay, with linear regression, shaded area is 95% confidence interval. (D) Percentage of eligible patients given lifestyle or medical advice. Smoking cessation is advised for smokers with polycythaemia secondary to hypoxia. Aspirin is recommended for idiopathic polycythaemia if indicated by cardiovascular risk profile, and guideline advises consider for congenital polycythaemia. (E) Total venesection procedures undergone by patients over the 5‐year study period. (F) Hct target values for venesection patients. BSH, British Society for Haematology; WHO, World Health Organization.

**TABLE 1:** Variables at diagnosis and outcome data for whole cohort, and according to venesection status.

Characteristics	All	Not venesected	Venesected	*p* ^a^
	*N* = 206	*N* = 123	*N* = 83	
Age at diagnosis^b^ (years)	54 (46, 65)	55 (46, 66)	53 (46, 64)	0.5
Female	61 (30%)	44 (36%)	17 (20%)	0.018
Male	145 (70%)	79 (64%)	66 (80%)	
Ethnic category (40 unknown)				>0.9
White	150 (90%)	90 (90%)	60 (91%)	
Asian	7 (4.2%)	4 (4.0%)	3 (4.5%)	
Other	5 (3.0%)	4 (4.0%)	1 (1.5%)	
Mixed	4 (2.4%)	2 (2.0%)	2 (3.0%)	
Polycythaemia diagnosis				0.12
Secondary (definite)	54 (26%)	28 (23%)	26 (31%)	
Secondary (probable)	101 (49%)	67 (54%)	34 (41%)	
Idiopathic	24 (12%)	10 (8.1%)	14 (17%)	
Apparent/relative	21 (10%)	13 (11%)	8 (9.6%)	
Congenital	1 (0.5%)	1 (0.8%)	0 (0%)	
Post‐transplant	2 (1.0%)	1 (0.8%)	1 (1.2%)	
None	3 (1.5%)	3 (2.4%)	0 (0%)	
Hct at diagnosis^b^	0.54 (0.52, 0.57)	0.52 (0.51, 0.54)	0.56 (0.54, 0.58)	<0.001
Underlying causes				
Smoking	115 (56%)	68 (55%)	47 (57%)	0.8
Sleep apnoea	23 (11%)	12 (9.8%)	11 (13%)	0.4
Chronic lung disease	40 (19%)	23 (19%)	17 (20%)	0.8
R → L cardiac shunt	3 (1.5%)	3 (2.4%)	0 (0%)	0.3
EPO use	1 (0.5%)	1 (0.8%)	0 (0%)	>0.9
Androgen use	11 (5.3%)	8 (6.5%)	3 (3.6%)	0.5
Diuretic use	14 (6.8%)	9 (7.3%)	5 (6.0%)	0.7
Alcohol	71 (34%)	37 (30%)	34 (41%)	0.11
Any neoplasm	4 (1.9%)	3 (2.4%)	1 (1.2%)	0.6
Renal pathology	9 (4.4%)	6 (4.9%)	3 (3.6%)	0.7
Red cell mass^b^ (nuclear; 162 unknown)	1.27 (1.13, 1.46)	1.17 (1.10, 1.44)	1.33 (1.14, 1.46)	0.3
Serum EPO (55 unknown)				0.086
Low	27 (18%)	10 (12%)	17 (25%)	
Normal	107 (71%)	64 (78%)	43 (62%)	
High	17 (11%)	8 (9.8%)	9 (13%)	
Alcohol intake (1 unknown)				0.084
No excess	134 (65%)	86 (70%)	48 (58%)	
Hazardous	48 (23%)	22 (18%)	26 (31%)	
Harmful	23 (11%)	14 (11%)	9 (11%)	
Respiratory comorbidity	71 (34%)	39 (32%)	32 (39%)	0.3
Cardiovascular comorbidity	112 (54%)	78 (63%)	34 (41%)	0.002
Total comorbidities^b^	1 (1, 2)	2 (1, 3)	1 (0, 2)	0.013
Prior arterial ischaemic event	32 (16%)	19 (15%)	13 (16%)	>0.9
Prior venous event	17 (8.3%)	8 (6.5%)	9 (11%)	0.3
Arterial event in follow‐up	16 (7.8%)	7 (5.7%)	9 (9.6%)	0.2
Venous event in follow‐up	6 (2.9%)	4 (3.3%)	2 (2.4%)	>0.9
Died in follow‐up	31 (15%)	23 (19%)	8 (9.6%)	0.074

Abbreviations: EPO, erythropoietin; Hct, haematocrit; R → L, right to left.

^a^ Wilcoxon rank sum test for continuous variables; Pearson's chi‐squared test or Fisher's exact test (where all expected values ≥5) for categorical variables.

^b^ Median (interquartile range) for continuous variables.

Regarding compliance with BSH guidelines recommendations, red cell mass and EPO levels were measured in 20% and 73% respectively (Figure 1B,C). The correlation between red cell mass and Hct was very weak (*R* ^2^ = 0.08). The majority of patients were given appropriate smoking, alcohol and androgen reduction advice (Figure 1D) but only 7% of eligible patients were started on aspirin (Figure 1D; Table S2). One or more (median 3) venesections were performed in 83 (40%) patients and 29% of patients undergoing >1 venesection had no target Hct documented (Figure 1E,F; Figures S1 and S2). Patients who underwent venesection were more likely to be male, have a higher Hct and have fewer comorbidities (Table 1).

Examining Hct over time, we found that the Hct was already reducing at the time of diagnosis (Figure 2A,B). Subsequently, the mean Hct fell by 0.04 (95% confidence interval [CI] 0.03–0.05, *p* < 0.0001) over the first year following diagnosis. At 5 years, mean Hct had fallen by 0.05 (95% CI: 0.04–0.06, *p* < 0.0001), irrespective of venesection treatments. Patients with persistent polycythaemia were more likely to be female and have high serum EPO than patients with variable polycythaemia (Table S1). After a median of 6.2 years of follow‐up, 2.9% of patients had a new venous thrombosis and 7.3% had a new arterial thrombosis/occlusion. The mortality rate was 15%. Interestingly, both freedom‐from‐thrombosis and mortality were unaffected by venesection or persistence of polycythaemia (Figure 2C,D; Figure S3). To eliminate immortal time bias, we performed a Cox proportional hazards analysis modelling venesection as a time‐dependent variable and again found no evidence of an association with thrombosis (hazard ratio: 0.88, 95% CI: 0.36–2.17, *p* = 0.79, Table S3). In a multivariable analysis of thrombosis, the only statistically significant (*p* < 0.05) association was with prior venous thrombosis (Table S4). There was no association with venesection when modelled as a time‐dependent variable.

**FIGURE 2:**
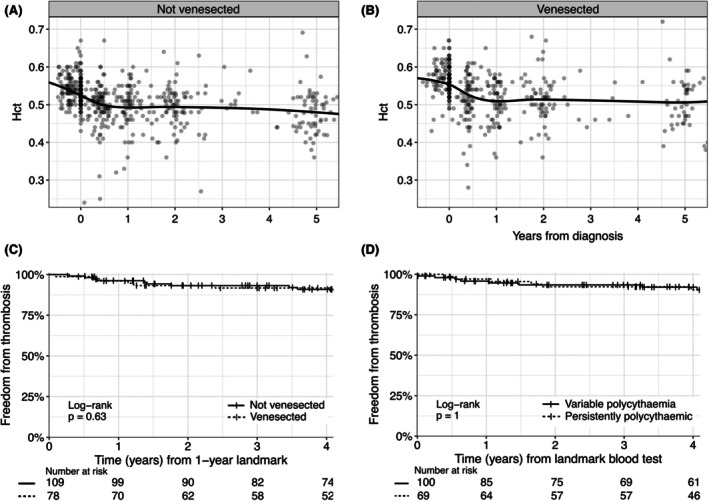
Prognosis of patients with secondary polycythaemia. Haematocrit (Hct) over time, with a plotted local polynomial regression line of fit, (A) for patients who were never venesected or (B) underwent one or more venesections (B). (C) Kaplan–Meier curves of freedom from thrombosis (death events are censored), comparing patients who never underwent venesection with those who were venesected at least once, starting at the 1 year landmark, as 87% of venesected patients had their first procedure within 12 months of diagnosis. Seven patients were excluded due to death prior to landmark, and 12 were lost to follow up prior to landmark. (D) Kaplan–Meier curves of freedom from thrombosis (death events censored), comparing patients with persistent polycythaemia with those whose Hct intermittently fell below the diagnostic threshold. Persistent polycythaemia is defined as Hct >0.48 (0.52 for males) prior to diagnosis, at diagnosis and at 6 and 12 months post‐diagnosis (or 24 months, if not done at 12 months). For this analysis, the landmark time = 0 is the 12‐month blood test (or if not available, the 24‐month blood test). Eight patients were excluded due to death prior to landmark, seven due to loss of follow‐up and 17 due to the absence of a 12‐ or 24‐month blood test result.

We examined the influence of venesection on Hct over time and freedom‐from‐thrombosis in subgroups affected by specific contributory factors and found similar patterns to those seen in the whole cohort (Figure S4).

Overall, in this 5‐year retrospective analysis of patients with secondary and idiopathic polycythaemia, we found variable adherence to BSH guidance in investigation and management. This variation in practice probably reflects the paucity of reliable evidence in this area. However, these data do provide some insight into the utility of some aspects of investigation and management. The correlation between Hct and red cell mass was extremely weak, and given the logistical challenges and cost, the role of red cell mass testing in secondary polycythaemia remains to be defined. The majority of patients were not venesected, which is in line with our international survey data.^15^ Patients who underwent venesection had a higher Hct and were 80% male, reflecting recommended venesection thresholds that are not sex‐specific. The influence of Hct on outcomes may be contingent on sex.^16^ Interestingly, we found that the mean Hct was falling at the time of diagnosis, perhaps related to prior intervention from referring clinicians and continued to fall in the first year after diagnosis, regardless of whether or not patients underwent venesection. This may represent reversion to the mean or the impact of advice and lifestyle interventions. The use of venesection was not associated with a reduced rate of thrombosis and nor was persistence of polycythaemia, which suggests that, in secondary polycythaemia, the raised Hct per se may not be a risk factor for thrombosis. We did not examine the effect of venesection on symptoms attributable to polycythaemia. Only 7% of potentially eligible patients were started on aspirin, and since the majority of thrombotic events were arterial, measures to promote appropriate antiplatelet use merit further consideration.

By its retrospective design, this study has some significant limitations. These include potential unmeasured baseline factors that influenced the decision to venesect which may have led to confounding, and incomplete ascertainment due to gaps in the medical record. 82% of patients were not tested for mutations in exon 12 of *JAK2*, so our cohort may include a small number of cases of PV. We did not collect data on SGLT2 inhibitor use, but the number of potentially affected patients is low, considering only 16/206 had type 2 diabetes and the limited availability of SGLT2 inhibitors in the UK in 2014–2018. Another limitation is our patient identification methodology using a *JAK2* test database. This yielded very few patients with congenital heart disease, so our results do not reflect this patient cohort. We were also not able to complete screening on nearly half the potentially eligible patients. Nonetheless, there was no systematic bias in patients screened, and this represents one of the largest recorded cohorts of secondary polycythaemia in the era of routine genetic testing. Internal validity was demonstrated by the finding that the only baseline factors significantly associated with increased thrombosis in univariate analyses were prior venous thrombosis and prior arterial thrombosis. We minimised immortal time bias by modelling venesection as a time‐dependent variable and using landmark analyses.

These results suggest that venesection for secondary/idiopathic polycythaemia may not affect thrombosis risk, and prospective studies focused on specific types of secondary polycythaemia are warranted. The use of venesection in this population is potentially harmful and has time and cost implications for health services and patients. Our results provide valuable baseline data on thrombosis rates, which could inform a randomised trial of venesection in patients with secondary polycythaemia. This could provide definitive evidence of the utility of venesection in secondary and idiopathic polycythaemia.

## AUTHOR CONTRIBUTIONS

BM conceived and designed the study, collected and analysed data and wrote and edited the manuscript. PLRN conceived and designed the study, collected data and wrote and edited the manuscript. EJ collected data, wrote and edited the manuscript. CM, KS, JP, MA, MB, CL, FN, SA, PD, HD, RL, MC, PR, MA, WB and TJ collected data and edited the manuscript. RJB conceived and designed the study, wrote and edited the manuscript. HaemSTAR Collaborators: Zoha Mansoor, Farhan Patel, Sanober Sanober, Rahul Ghandi and Eman Hassan collected data.

## FUNDING INFORMATION

No funding was received to support this work.

## CONFLICT OF INTEREST STATEMENT

No authors declare any conflicts of interest.

## ETHICS APPROVAL STATEMENT

This audit was approved by the audit departments of University Hospitals Birmingham NHS Foundation Trust and University Hospitals of North Midlands NHS Trust.

## PATIENT CONSENT STATEMENT

Patient consent was not required for this retrospective audit.

## Supporting information


Data S1.

## Data Availability

The corresponding authors will share anonymised audit data with colleagues if reasonable requests are made.
