# Supplementary material for: Venesection and resolution of erythrocytosis are not associated with reduced thrombotic risk in secondary and idiopathic polycythaemia: Results from a dual centre, 5‐year retrospective study
Source: Br J Haematol. 2025 Jun 30;207(3):1127–32. doi: 10.1111/bjh.20235 (PMC12436238; doi:10.1111/bjh.20235)
Supplement: Supplementary file 1 — Data S1. [file BJH-207-1127-s001.docx]

SUPPLEMENTARY DATA

Venesection and resolution of erythrocytosis are not associated with reduced thrombotic risk in secondary and idiopathic polycythaemia. Results from a dual centre, 5-year retrospective study.

Maybury *et al.* 2025

**METHODS**

Patients with a negative *JAK2* V617F test result between 2014 and 2018 were identified from a central genetics laboratory, and screened for polycythaemia at the time of genetic testing. Inclusion criteria were a haematocrit (Hct) above 0.48 (for females) or 0.49 (for males) as per WHO criteria; absence of any myeloproliferative neoplasm; first investigated between 2014-2018. Data were collected retrospectively using REDCap electronic data capture tools hosted at University of Birmingham, UK.^1^ Analyses were performed in R 4.4.2 using the packages survival, survminer, tidyverse, vtable, gtsummary, forestmodel, expss and ggtext. For venesection comparisons, patients who underwent any venesection were assigned in the ‘venesection’ group. For comparisons based on persistence of polycythaemia, patients who had Hct >0.48 (females) or >0.52 (males) at all time points (where a result was available) from 3 months prior to diagnosis up to 12 months post-diagnosis, or, where the 12 month time point was not available, until 24 months post-diagnosis, were considered to be persistently polycythaemic. All other patients who had a Hct result at either 12 or 24 months post-diagnosis were assigned to the ‘variable polycythaemia’ group. For this landmark analysis, the t=0 for each patient was the date of the 12 month Hct result if available, or if not, the date of the 24 month Hct result. For multiple events Cox analyses, the Andersen & Gill extension of Cox’s model was used.^2^ No corrections were made for multiple hypothesis testing.

**RESULTS**

| **Characteristic** | **Variable polycythaemia**  N = 116*^1^* | **Persistently polycythaemic**  N = 53*^1^* | | | | **p-value***^2^* |  |
| --- | --- | --- | --- | --- | --- | --- | --- |
| Age at diagnosis (years)* | 54 (45, 66) | 58 (48, 66) | | | | 0.7 |  |
| Female | 31 (27%) | 23 (43%) | | | | 0.031 |  |
| Male | 85 (73%) | 30 (57%) | | | |  |  |
| Ethnic category (14 unknown) |  |  | | | | 0.3 |  |
| White | 91 (89%) | 37 (95%) | | | |  |  |
| Asian | 7 (6.9%) | 0 (0%) | | | |  |  |
| Other | 2 (2.0%) | 1 (2.6%) | | | |  |  |
| Mixed | 2 (2.0%) | 1 (2.6%) | | | |  |  |
| Erythrocytosis diagnosis |  |  | | | | 0.3 |  |
| Secondary (definite) | 31 (27%) | 19 (36%) | | | |  |  |
| Secondary (probable) | 55 (47%) | 23 (43%) | | | |  |  |
| Idiopathic | 14 (12%) | 6 (11%) | | | |  |  |
| Apparent/relative | 14 (12%) | 2 (3.8%) | | | |  |  |
| Congenital | 0 (0%) | 1 (1.9%) | | | |  |  |
| Post-transplant | 1 (0.9%) | 1 (1.9%) | | | |  |  |
| None | 1 (0.9%) | 1 (1.9%) | | | |  |  |
| Hct at diagnosis* | 0.54 (0.51, 0.57) | 0.54 (0.52, 0.56) | | | | 0.7 |  |
| Hct 1 yr later (22 unknown)* | 0.50 (0.46, 0.54) | 0.53 (0.50, 0.55) | | | | 0.004 |  |
| Hct 5 yrs later (48 unknown)* | 0.47 (0.44, 0.50) | 0.54 (0.52, 0.57) | | | | <0.001 |  |
| Underlying causes |  |  | | | |  |  |
| Smoking | 66 (57%) | 28 (53%) | | | | 0.6 |  |
| Sleep apnoea | 13 (11%) | 7 (13%) | | | | 0.7 |  |
| Chronic lung disease | 19 (16%) | 15 (28%) | | | | 0.073 |  |
| R->L cardiac shunt | 2 (1.7%) | 1 (1.9%) | | | | >0.9 |  |
| EPO use | 1 (0.9%) | 0 (0%) | | | | >0.9 |  |
| Androgen use | 7 (6.0%) | 1 (1.9%) | | | | 0.4 |  |
| Diuretic use | 5 (4.3%) | 7 (13%) | | | | 0.052 |  |
| Alcohol | 46 (40%) | 13 (25%) | | | | 0.056 |  |
| Any neoplasm | 3 (2.6%) | 0 (0%) | | | | 0.6 |  |
| Renal pathology | 5 (4.3%) | 3 (5.7%) | | | | 0.7 |  |
| Red cell mass (nuclear;  127 unknown)* | 1.24 (1.12, 1.40) | 1.35 (1.20, 1.48) | | | | 0.3 |  |
| Serum EPO (41 unknown) |  |  | | | | 0.004 |  |
| Low | 18 (21%) | 5 (12%) | | | |  |  |
| Normal | 65 (75%) | 26 (63%) | | | |  |  |
| High | 4 (4.6%) | 10 (24%) | | | |  |  |
| Alcohol intake (1 unknown) |  |  | | | | 0.4 |  |
| No excess | 71 (62%) | 37 (70%) | | | |  |  |
| Hazardous | 28 (24%) | 12 (23%) | | | |  |  |
| Harmful | 16 (14%) | 4 (7.5%) | | | |  |  |
| Respiratory comorbidity | 36 (31%) | 22 (42%) | | | | 0.2 |  |
| Cardiovascular comorbidity | 68 (59%) | 31 (58%) | | | | >0.9 |  |
| Total comorbidities* | 1 (1, 2) | 1 (1, 3) | | | | 0.6 |  |
| Prior arterial ischaemic event | 22 (19%) | 5 (9.4%) | | | | 0.12 |  |
| Prior venous event | 10 (8.6%) | 4 (7.5%) | | | | >0.9 |  |
| On anticoagulant at diagnosis | 13 (11%) | 5 (9.4%) | | | | 0.7 |  |
| On antiplatelet at diagnosis | 30 (26%) | 15 (28%) | | | | 0.7 |  |
| Venesection intended | 34 (29%) | 23 (43%) | | | | 0.072 |  |
| Venesections performed |  |  | | | | 0.041 |  |
| Not venesected | 72 (62%) | 24 (45%) | | | |  |  |
| Venesected | 44 (38%) | 29 (55%) | | | |  |  |
| Arterial event in follow-up | 9 (7.8%) | 6 (11%) | | | | 0.6 |  |
| Venous event in follow-up | 4 (3.4%) | 1 (1.9%) | | | | >0.9 |  |
| Died in follow-up | 11 (9.5%) | 10 (19%) | | | | 0.086 |  |
| * Median (interquartile range) for continuous variables. | | |  |  |  |  |  |
| *^1^* Wilcoxon rank sum test for continuous variables; Pearson’s Chi-squared test or Fisher’s exact test (where all expected values ≥5) for categorical variables | | |  |  |  |  |  |

**Table S1.** Variables at diagnosis and outcome data according to the persistence of polycythaemia. Patients were considered persistently polycythaemic if Hct measurements between 3 months pre-diagnosis and 12 months post-diagnosis (or 24 months, if none at 12 months), where done, were >0.48 (females) or >0.52 (males). EPO: erythropoietin, Hct: haematocrit, R->L: right to left.

| **Audit standard** | **Number** |
| --- | --- |
| Of patients with secondary polycythaemia due to chronic lung disease or left->right shunt, who smoke, how many received smoking cessation advice? | 15 / 18 (83%) |
| Of current smokers with secondary polycythaemia due to chronic lung disease, left->right shunt or smoking, how many received smoking cessation advice? | 69 / 82 (84%) |
| Of patients with idiopathic polycythaemia, not already on antithrombotic medication, what proportion were advised to start aspirin? | 1 / 17 (5%) |
| Of patients with congenital polycythaemia, not already on antithrombotic medication, what proportion were advised to start aspirin? | 0 / 1 (0%) |

**Table S2.** Compliance with BSH guideline recommendations on non-venesection interventions. Guideline recommendations (grade of recommendation in brackets) are as follows: for secondary erythrocytosis due to chronic hypoxic pulmonary disease, smokers ‘should be strongly advised to stop’; for idiopathic erythrocytosis, aspirin is recommended ‘if otherwise clinically indicated’ (grade 1B) and, for congenital erythrocytosis, ‘consider low-dose aspirin’ (grade 2C). We did not collect data to verify whether patients with secondary erythrocytosis were hypoxic, and report results for smokers too.

**Figure S1.** Venesection target Hct for patients with idiopathic polycythaemia planned to undergo venesection. For idiopathic erythrocytosis BSH recommend venesection in selected cases, with an ‘arbitrary target Hct of <0.55’ or a target of <0.45 for those with thrombosis related to the erythrocytosis.

**Figure S2.** Target Hct for patients with secondary polycythaemia due to chronic lung disease or left-right shunt, planned to undergo venesection. For polycythaemia secondary to hypoxic pulmonary disease, BSH recommend venesection to a target of 0.5-0.52 for those with viscosity symptoms or Hct >0.56 otherwise.

**Androgen cases**

We identified 11 cases in which androgen use was a contributing factor. This was the sole contributing factor in 4, and in the remaining 7, smoking and/or sleep apnoea were also contributing factors. In the 4 cases where the underlying indication was available, testosterone supplementation was used as anabolic lifestyle supplement in 1 patient, and for insufficiency of pituitary or gonadal function in 3 patients. 1 had previous arterial thrombosis, none had previous venous thrombosis. Of the 11 patients where androgen (mis)use was thought to be contributing to their polycythaemia, 6 (55%) were advised to reduce this. The BSH make no specific recommendation regarding this, Urology^3^ and Endocrine^4^ society guidelines advise dose reductions where there is secondary polycythaemia.

**Congenital cases**

Only one case of congenital polycythaemia met BSH criteria for diagnosis of secondary polycthaemia, and no genetic cause was identified in this case. A second case met WHO criteria only, and in this case the polycythaemia was attributed to heterozygosity for Haemoglobin D. A third case of high affinity haemoglobin was identified during screening (Hb Andrew-Minneapolis) but this patient had been first investigated prior to our inclusion window, and so was not eligible for the study.

**Cancer cases**

6/206 patients had an active cancer at the time of diagnosis: one each of lung, head & neck, prostate, lymphoma, other solid organ, and other haematological. Of these 6, 3 were on treatment. An additional 8 patients had a prior history of cancer, in remission at time of polycythaemia diagnosis.

A B

**Figure S3.** Kaplan-Meier plots of overall survival. (A) According to venesection status. The landmark timepoint is 1 year post-diagnosis, to minimise immortal time bias. 7 patients were excluded due to death prior to landmark, and 12 were lost to follow up prior to landmark. (B) According to persistent polycythaemia, defined as Hct > .48 (.52 for males) prior to diagnosis, at diagnosis, and at 6 and 12 months post-diagnosis (or 24 months, if not done at 12 months). For this analysis the landmark time=0 is the 12 month blood test (or if not available, the 24 month blood test). 8 patients were excluded due to death prior to landmark, 7 due to loss of follow up, and 17 due to the absence of a 12 or 24 month blood test result.

| **Variable** | **Hazard ratio** | **95% confidence interval** | **p** |
| --- | --- | --- | --- |
| Age per increment of 1 year | 1.01 | 0.96 - 1.06 | 0.81 |
| Male sex (vs female) | 1.09 | 0.37 - 3.22 | 0.88 |
| Hct at diagnosis (per 0.1 increment) | 1.30 | 0.50 - 3.37 | 0.59 |
| Nuclear red cell mass ratio (per 0.1 increment) | 0.95 | 0.79 - 1.14 | 0.59 |
| Platelet count at diagnosis per increment of 1 | 0.99 | 0.99 - 1.01 | 0.70 |
| Prior arterial event (vs none) | 3.49 | 1.05 - 11.5 | 0.04 |
| Prior venous event (vs none) | 4.32 | 1.48 - 12.7 | 0.01 |
| Active cancer (vs none) | 3.09 | 0.71 - 13.4 | 0.13 |
| Any cardiovascular morbidity (vs none) | 1.36 | 0.47 - 3.96 | 0.57 |
| Venesection (time-dependent) (vs none) | 0.88 | 0.36 - 2.17 | 0.79 |

**Table S3.** Univariate Cox proportional hazard analyses. In these models, thrombosis (arterial or venous) is an event which can occur multiple times.

| **Variable** | **Hazard ratio** | **95% confidence interval** | **p** |
| --- | --- | --- | --- |
| Prior arterial event (vs none) | 3.04 | 0.79 – 11.7 | 0.11 |
| Prior venous event (vs none) | 4.26 | 1.10 – 16.5 | 0.04 |
| Active cancer (vs none) | 3.42 | 0.57 – 20.5 | 0.18 |
| Venesection (time-dependent) (vs none) | 0.80 | 0.31 – 2.09 | 0.65 |

**Table S4.** Multivariate Cox proportional hazard analysis for thrombosis. Variables with a p-value >0.5 in univariate analysis were excluded, with the exception of venesection (as the variable of interest). In this model, thrombosis (arterial or venous) is an event which can occur multiple times.


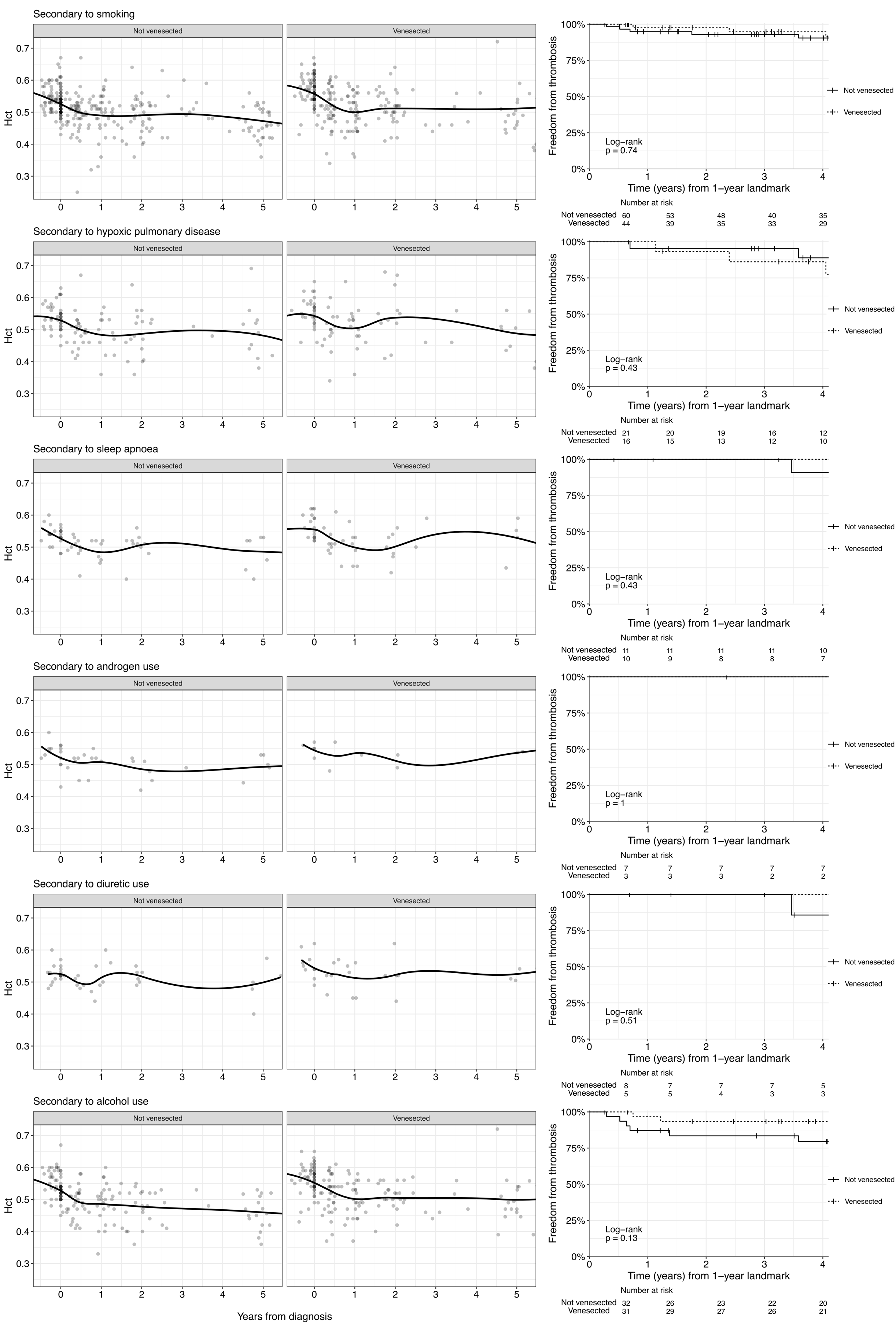


**Figure S4.** Effect of venesection according to underlying causes of polycythaemia. Hct over time (left), with a plotted local polynomial regression line of fit, for patients who were never venesected or underwent 1 or more venesections. Kaplan-Meier curves (right) of freedom from thrombosis (death events are censored), comparing patients who never underwent venesection with those who were venesected at least once, starting at the 1 year landmark. Only underlying causes with >5 affected patients are shown.

**REFERENCES:**

1. Harris PA, Taylor R, Minor BL, Elliott V, Fernandez M, O’Neal L, et al. The REDCap consortium: Building an international community of software platform partners. *Journal of Biomedical Informatics*. 2019; **95**:103208.

2. Andersen PK, Gill RD. Cox’s Regression Model for Counting Processes: A Large Sample Study. *The Annals of Statistics*. 1982; **10**(4):1100–20.

3. Minhas S, Bettocchi C, Boeri L, Capogrosso P, Carvalho J, Cilesiz NC, et al. European Association of Urology Guidelines on Male Sexual and Reproductive Health: 2021 Update on Male Infertility. *European Urology*. 2021; **80**(5):603–20.

4. Hembree WC, Cohen-Kettenis PT, Gooren L, Hannema SE, Meyer WJ, Murad MH, et al. Endocrine Treatment of Gender-Dysphoric/Gender-Incongruent Persons: An Endocrine Society* Clinical Practice Guideline. *The Journal of Clinical Endocrinology & Metabolism*. 2017; **102**(11):3869–903.
